# nurP28, a New-to-Nature Zein-Derived Peptide, Enhances the Therapeutic Effect of Docetaxel in Breast Cancer Monolayers and Spheroids

**DOI:** 10.3390/molecules27092824

**Published:** 2022-04-29

**Authors:** Plinio Alejandro Trinidad-Calderón, Laura Margarita López-Castillo, Salvador Gallegos-Martínez, Grissel Trujillo-de Santiago, Silverio García-Lara, Mario Moisés Álvarez

**Affiliations:** 1 Tecnológico de Monterrey, Escuela de Ingeniería y Ciencias, Ave. Eugenio Garza Sada 2501, Monterrey 64849, Mexico; a00822365@tec.mx (P.A.T.-C.); ml@indexalimentos.com.mx (L.M.L.-C.); a00822384@tec.mx (S.G.-M.); grissel@tec.mx (G.T.-d.S.); sgarcialara@tec.mx (S.G.-L.); 2 InDeX Innovación y Desarrollo en Alimentos, Av. Lázaro Cárdenas 4336, Monterrey 64930, Mexico

**Keywords:** nurP28, zein, derived, peptide, docetaxel, breast, cancer, spheroid, therapeutic

## Abstract

The development of novel cancer therapeutic strategies has garnered increasing interest in cancer research. Among the therapeutic choices, chemosensitizers have shown exciting prospects. Peptides are an attractive alternative among the molecules that may be used as chemosensitizers. We rationally designed a new-to-nature peptide, nurP28, derived from the 22-kDa α-zein protein sequence (entry Q00919_MAIZE). The resultant sequence of the nurP28 peptide after the addition of arginine residues was LALLALLRLRRRATTAFIIP, and we added acetyl and amide groups at the N- and C-terminus, respectively, for capping. We evaluated the cytotoxicity of the nurP28 peptide alone and in combination with docetaxel in fibroblast monolayers and breast cancer monolayers and spheroids. Our results indicated that nurP28 is not cytotoxic to human fibroblasts or cancer cells. Nevertheless, when combined with 1 µM docetaxel, 3 ng/mL nurP28 induced equivalent (in MCF7 monolayers) and higher (in MCF7 spheroids) cytotoxic effects than 10-fold higher doses of docetaxel alone. These findings suggest that nurP28 may act as a chemosensitizer in breast cancer treatment. This study describes the enhancing “anti-cancer” effects of nurP28 in breast cancer 2D and 3D cultures treated with docetaxel. Further studies should explore the mechanisms underlying these effects and assess the clinical potential of our findings using animal models.

## 1. Introduction

Breast cancer is the most widely diagnosed cancer [1], accounting for 11.7% of cases worldwide [2]. Nearly 81.4% of patients diagnosed with breast cancer receive chemotherapy [3]. However, currently available chemotherapeutic drugs have limitations in terms of their efficacy and elicit noteworthy side effects, such as alopecia, bradycardia, and nausea [4].

Demand has been growing for novel therapeutic options to treat cancer [5,6,7], including agents to potentiate the effect of known “anticancer” drugs [8]. Among the numerous therapeutic choices available, chemosensitizers have shown exciting prospects [9]. Anticancer chemosensitizers increase the effectiveness of certain drugs by lowering the doses required to achieve the same therapeutic effect [10]. This may attenuate the side effects of chemotherapy [11].

Peptides are an attractive alternative among the spectrum of molecules that may be used as chemosensitizers [12,13,14]. Peptides, which are polymers containing <40 amino acid residues [15], have lately been explored for their therapeutic potential [2]. These molecules have piqued the interest of the pharmaceutical industry, with some even reaching clinical trials [16,17].

Numerous studies have highlighted the importance of peptides in cancer therapy, including their intrinsic antitumor effect [2,18] and their ability to facilitate the transport of other molecules (e.g., anticancer drugs) inside cells [19,20]. Furthermore, peptides have advantages over molecules with a higher molecular weight [21], mainly in terms of feasibility to be synthesized [22], minimal toxicity to normal cells [23], and potential to improve penetration of other molecules into solid tumor tissues [2].

Here, we explore the potential of nurP28, an engineered peptide derived from native α-zein (the main constitutive protein of maize), to enhance the activity of docetaxel (DTX) in breast cancer monolayers (two-dimensional (2D) culture) and spheroids (three-dimensional (3D) culture), in combination with DTX.

Different strategies have been experimentally tested to alleviate these problems, including the encapsulation of DTX in micelles, the development of nanoparticles loaded with DTX, and the engineering of strategies for the co-delivery of DTX with diverse other compounds [24,25,26]. Recently, conjugates of DTX and peptides have been developed for the treatment of breast cancer [27].

DTX, a targeting cancer drug from the taxane family [28,29], is commonly used to treat localized or metastatic breast cancer due to its efficacy in a wide range of cancer types [30]. DTX is administered intravenously, alone or in combination with other therapeutic agents [31]. As with many other frequently used anti-cancer drugs, DTX has limitations. For example, DTX has poor water solubility [32] and a wide range of undesirable side effects [33] that are associated with its cytotoxicity. In addition, patients frequently develop DTX resistance after prolonged use [34].

To our knowledge, the present paper is the first to report a chemo-sensitizing effect of maize-derived peptides of known sequence in cancer cells [35].

## 2. Results

### 2.1. Design of nurP28 Peptide

**Figure 1:**
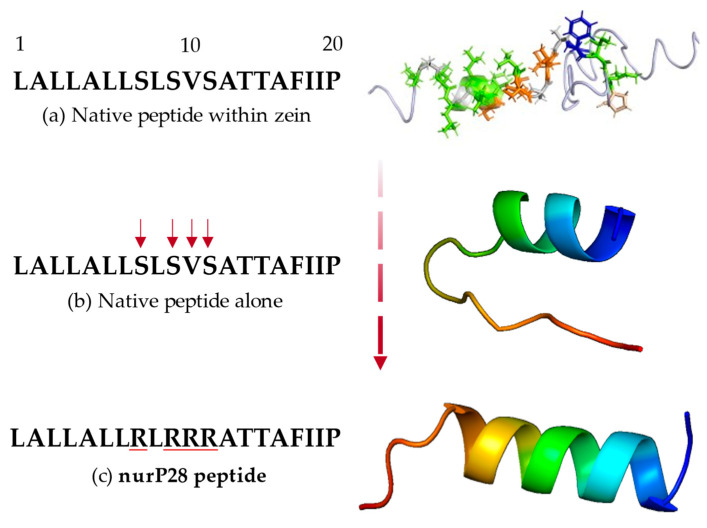
Design steps and molecular structure of nurP28 peptide. (**a**) Native peptide within zein protein; (**b**) native peptide alone showing the sequence unsuitable for synthesis; and (**c**) nurP28, the resulting modified peptide. Amino acid residues are colored according to the RasMol scheme within the zein structure. In each structure, red and blue ends indicate the C- and N-terminus of the represented peptide, respectively.

To define the sequence of the nurP28 peptide, we performed an in silico analysis of the 22-kDa α-zein protein sequence (entry Q00919_MAIZE) using the TMHMM Server v.2.0, [36] and PROTEUS Structure Prediction Server 2.0 [37]. We observed a potential transmembrane sequence within the 22-kDa α-zein (LALLALLSLSVSATTAFIIP) (Figure 1a), and owing to its neutral charge, it was found to be unsuitable for synthesis as a native peptide.

As a result, we rationally modified the native sequence by adding positively charged amino acid residues to create our designated nurP28 peptide; in particular, we modified the amino acids Ser8, Ser10, Val11, and Ser12 to arginine residues in the native sequence (Figure 1b).

After adding the arginine residues, the resultant sequence of the nurP28 peptide was LALLALLRLRRRATTAFIIP. The nurP28 peptide contained 20 amino acids corresponding to leucine (30%), alanine (20%), arginine (20%), isoleucine (10%), threonine (10%), proline (5%), and phenylalanine (5%). In terms of polarity, 70% of the amino acids in the nurP28 peptide were nonpolar, 20% were polar and ionizable, and 10% were polar and uncharged. This composition yielded a net charge of +4.

Moreover, the native nurP28 peptide exhibited a linear domain proximal to the N-terminus (Figure 1b) and a double α-helix structure proximal to the C-terminus. The structure of the nurP28 peptide was drastically altered when Ser8, Ser10, Val11, and Ser12 were replaced with arginine residues—an ionizable polar amino acid (Figure 1c). The resulting secondary structure exhibited 3.5 full α-helices (Video S1).

We also added acetyl and amide groups to the N- and C-terminus, respectively, for capping to obtain the final peptide sequence of Ac-LALLALLRLRRRATTAFIIP-NH_2_.

### 2.2. Cytocompatibility of nurP28 Peptide

We tested the cytocompatibility of the nurP28 peptide on BJ fibroblasts. Briefly, we exposed the fibroblasts to different concentrations of nurP28 peptide and/or DTX, one of the most commonly used chemotherapeutic drugs for breast cancer.

**Figure 2:**
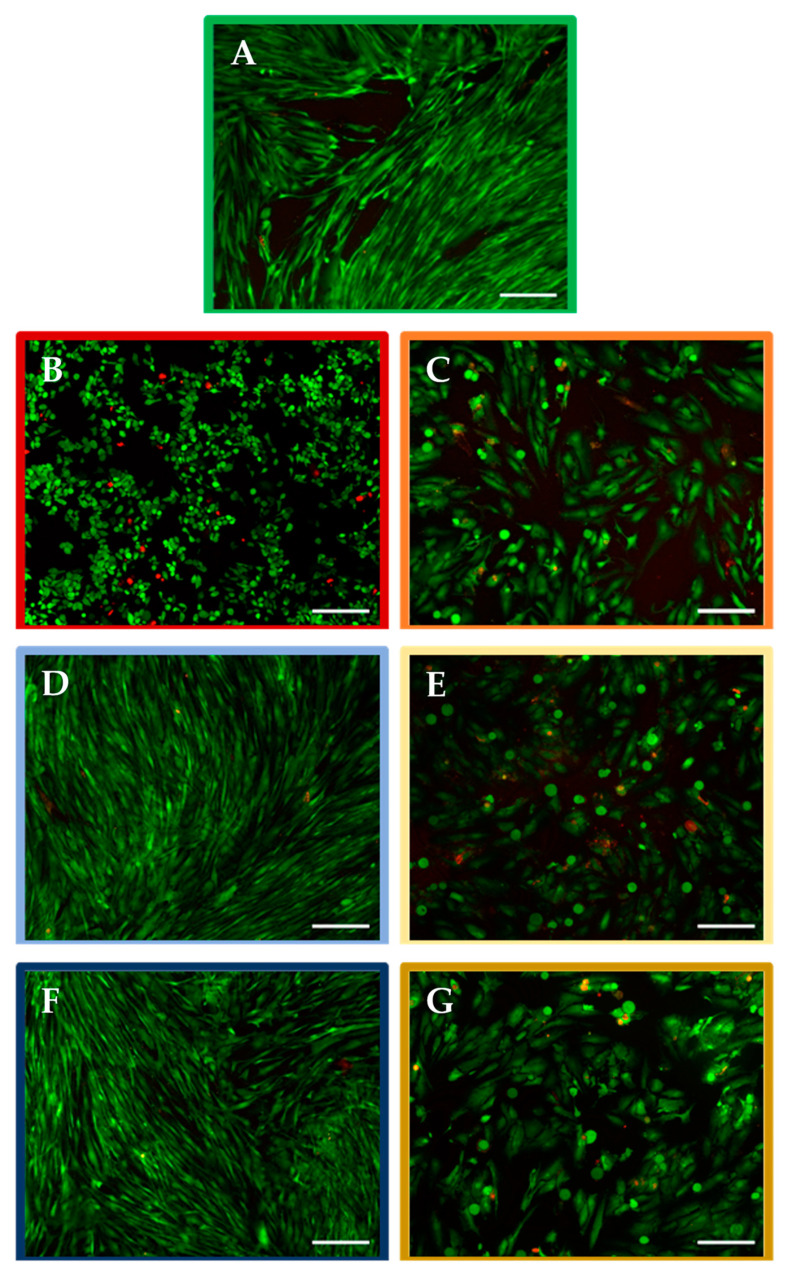
Live/dead images of human BJ fibroblasts after 48 h of exposure to nurP28 peptide alone and in combination with DTX. (**A**) Untreated cells (vehicle control); (**B**) 10 µM DTX; (**C**) 1 µM DTX; (**D**) 3 ng/mL nurP28; (**E**) 3 ng/mL nurP28 + 1 µM DTX; (**F**) 300 ng/mL nurP28; and (**G**) 300 ng/mL nurP28 + 1 µM DTX. Viable cells appear green and those with compromised metabolic activity appear orange/red. Magnification: 10×. Scale bar: 100 µM.

Figure 2 shows the representative images of live/dead assays conducted using BJ fibroblasts exposed to different concentrations of nurP28 and/or DTX for 48 h.

In contrast, cultures exposed to 10 µM DTX showed signs of membrane blebbing. Likewise, cells treated with 10 µM DTX seemed rounder than the control cells.

Similarly, BJ cultures exposed to 1 µM DTX demonstrated changes in cell density and morphology—the cells exhibited a rounded shape and the number of cells was less compared with the control. Moreover, signs of membrane blebbing were observed in BJ cells exposed to 1 µM DTX.

Interestingly, we did not observe any evidence of cytotoxicity in BJ cultures exposed to 3 ng/mL and 300 ng/mL nurP28 alone. In these cultures, highly confluent monolayers of viable and elongated cells were observed.

The combination treatments (3 ng/mL nurP28 and 300 ng/mL nurP28 + 1 µM DTX) induced morphological changes similar to those in cells exposed to 1 µM DTX. However, the combination of 3 ng/mL nurP28 + 1 µM DTX was more effective than 1 µM DTX in inducing changes in morphology (rounded cells) and cell density (fewer cells).

**Figure 3:**
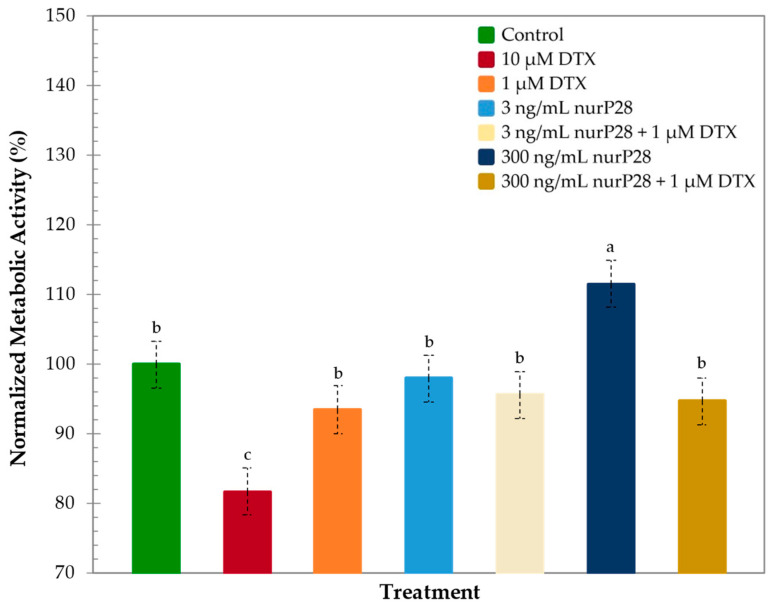
Normalized metabolic activity of human BJ fibroblasts after 48 h of exposure to nurP28 peptide alone and in combination with DTX. Results expressed as mean ± SD (*n* = 3). The letters a, b, and c indicate a statistically significant difference at *p* < 0.01. Bars with no common letters are significantly different.

Subsequently, we quantitatively assessed and normalized the metabolic activities (nMA) observed in each of the treatment groups in comparison with those observed in the control group (Figure 3).

Apparent differences between BJ cells exposed to different concentrations of DTX and nurP28 were observed. Control cells (without DTX or nurP28) exhibited normal cell growth, and confluent layers of elongated cells with an intact membrane were observed.

In this regard, metabolic activity was lower in BJ cells treated with 10 µM DTX (81.7% nMA) than in the control cells. Likewise, metabolic activity was higher in BJ cells treated with 1 µM DTX (93.4% nMA) than in those treated with 10 µM DTX. These data were statistically similar to the control.

Interestingly, no significant differences were observed in the metabolic activities of BJ cells treated with the combined therapies of 1 µM DTX + 3 ng/mL and 300 ng/mL nurP28 (95.5% and 94.6% nMA, respectively), those treated with 3 ng/mL nurP28 alone, and control (without nurP28 and DTX).

Although treatment with 3 ng/mL nurP28 also showed a similar trend (97.9% nMA) compared with the control, 300 ng/mL nurP28 induced significantly higher metabolic activity (111.5% nMA) compared with the control.

These results suggested that nurP28 is not cytotoxic to human fibroblasts within the range of the tested concentrations (i.e., 3 and 300 ng/mL).

### 2.3. Chemosentitizing Ability of nurP28 Peptide against Breast Cancer Cells in 2D Culture

**Figure 4:**
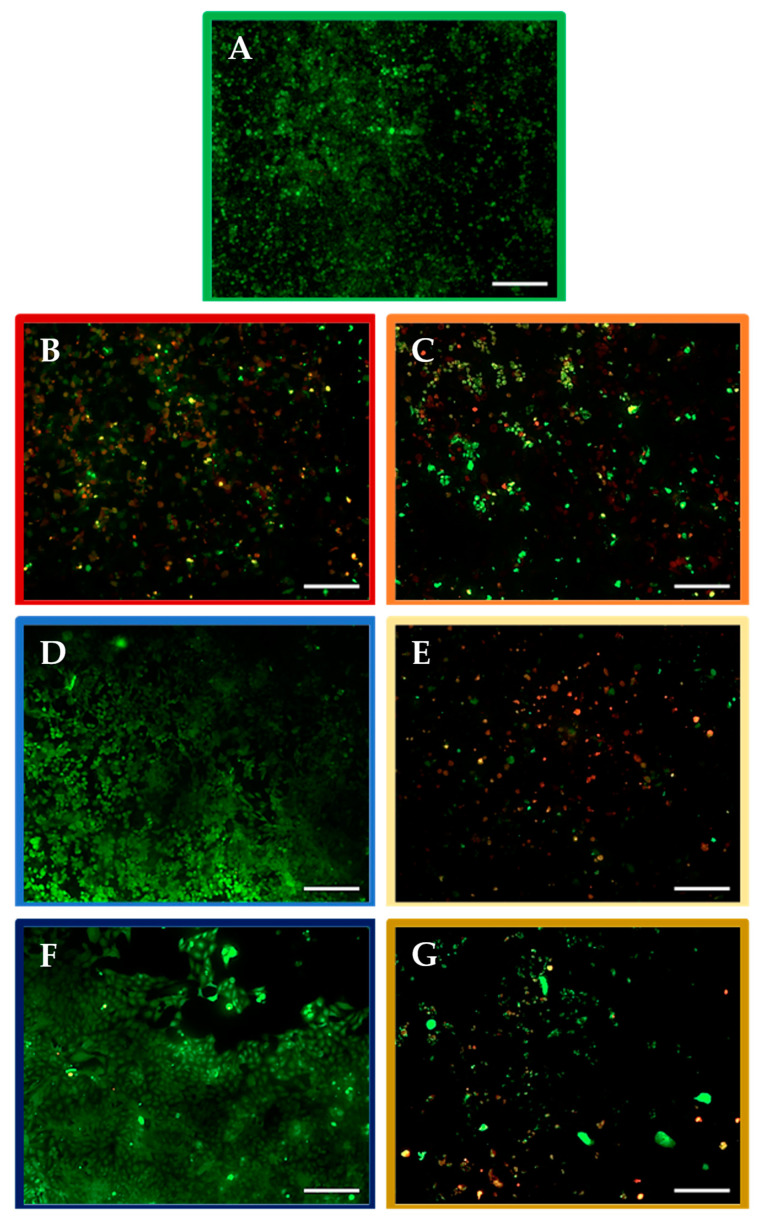
Images of live/dead assays conducted using breast cancer MCF7 cell monolayers after 48 h exposure to nurP28 peptide alone and in combination with DTX. (**A**) 10 µM DTX; (**B**) 1 µM DTX; (**C**) 3 ng/mL nurP28; (D) 3 ng/mL nurP28 + 1 µM DTX; (**E**) 300 ng/mL nurP28; (**F**) 300 ng/mL nurP28 + 1 µM DTX; and (**G**) untreated cells (vehicle control). Viable cells appear green and those with compromised metabolic activity appear orange/red. Magnification: 10×. Scale bar: 200 µM.

We evaluated the chemo-sensitizing potential of the nurP28 peptide in MCF7 cancer cell monolayers. Figure 4 depicts the representative images of live/dead assays conducted using MCF7 monolayer cultures after 48 h of exposure to different concentrations of the nurP28 peptide and/or DTX.

We observed differences among the MCF7 cells treated with DTX and nurP28. Control (without DTX or nurP28) cells were confluent, greater in number, and showed normal cell growth. In comparison, cells treated with 10 µM DTX showed signs of membrane blebbing and multiple cell death events.

Treatment with 1 µM DTX resulted in changes in cell density and morphology in MCF7 cultures—the number of cells in cultures treated with 1 µM DTX was lower in comparison with that in the control cultures. Nevertheless, this decrease was not as great as that observed in cultures treated with 10 µM DTX. Additionally, we observed some cells undergoing cell death.

In contrast, treatment with 3 ng/mL and 300 ng/mL nurP28 did not induce cytotoxicity. Moreover, the cultures treated with 3 ng/mL nurP28 showed more confluent viable cells than the control cultures.

The combination treatment with 3 ng/mL nurP28 and 1 µM DTX induced a response similar to treatment with 10 µM DTX, with evidence of membrane blebbing and multiple cell death events. Nevertheless, cultures treated with 300 ng/mL nurP28 + 1 µM DTX had much fewer cells than those treated with any other treatment.

**Figure 5:**
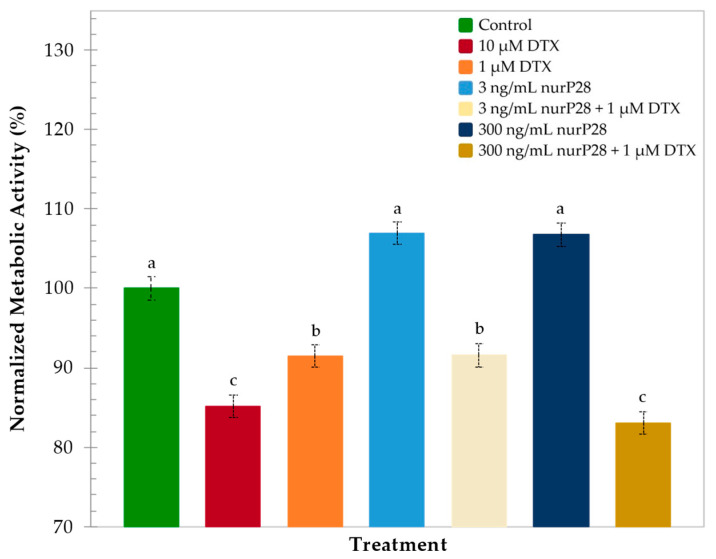
Normalized metabolic activity of breast cancer MCF7 monolayer cells after 48 h of exposure to nurP28 peptide alone and in combination with DTX. Results expressed as mean ± SD (*n* = 3). The letters a, b and c indicate a statistically significant difference at *p* < 0.01. Bars with no common letters are significantly different.

Considering these findings, we assayed the metabolic activity of the treated breast cancer monolayer cells (Figure 5).

In general, we observed high metabolic activities in the control (without DTX or nurP28) and the cultures exposed to nurP28 alone, suggesting that nurP28 administered as a monotherapy enhances the growth of MCF7 cells, probably by functioning as a protein nutrient.

Compared with the control, cell viability was lower in the cultures treated with DTX and the combination treatment. The statistical analysis of the metabolic activity values discerned three significantly different response groups. The first group included 3 ng/mL and 300 ng/mL nurP28 peptide and the control. The second group included 1 µM DTX and 3 ng/mL nurP28 + 1 µM DTX, exhibiting normalized metabolic activity slightly above 90%. The third group included 10 µM DTX (85.1% nMA) and 300 ng/mL nurP28 + 1 µM DTX (83.0% nMA).

In the last group, 300 ng/mL nurP28 demonstrated the ability to potentiate the effect of 1 µM DTX and induce a cytotoxic response equivalent to that induced by a 10-fold higher concentration of DTX alone.

### 2.4. Chemosentitizing Ability of nurP28 Peptide in 3D Breast Cancer Spheroids

**Figure 6:**
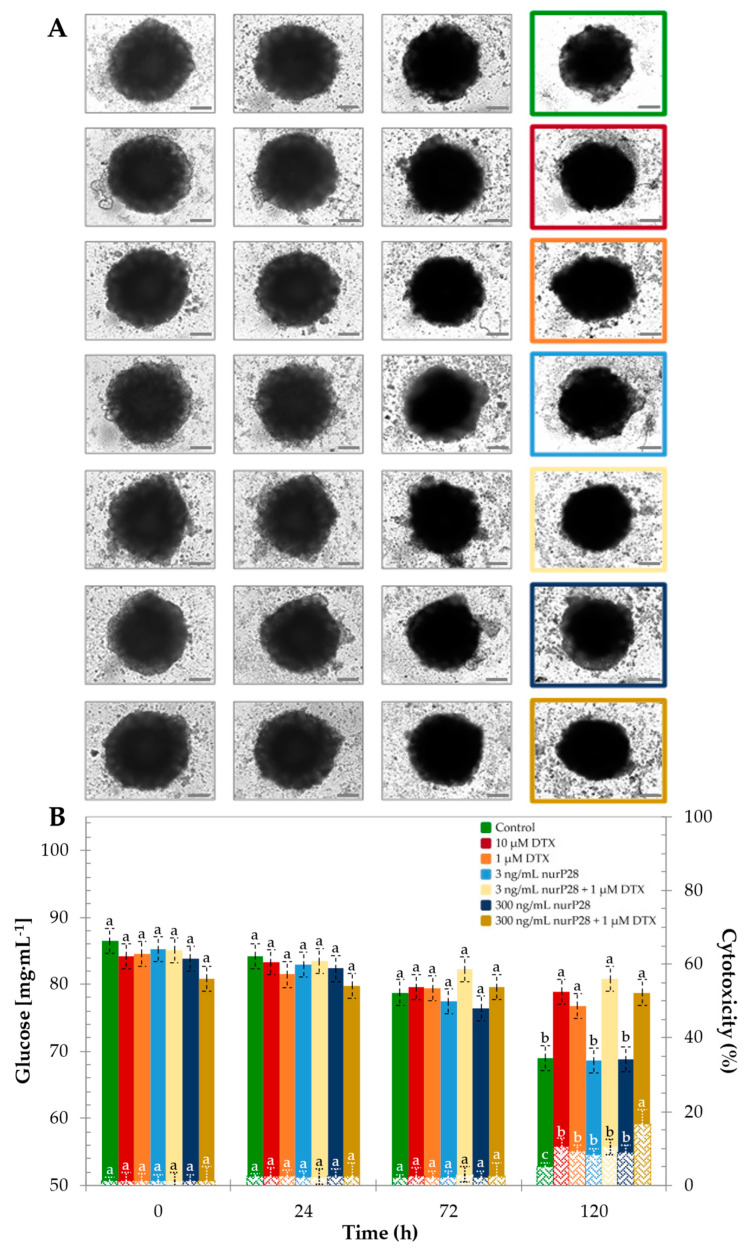
Monitoring breast cancer MCF7 spheroids treated with nurP28 peptide alone and in combination with DTX at 0, 24, 72, and 120 h. (**A**) Bright-field images of MCF7 spheroids exposed to the different treatments; (**B**) Glucose concentration (primary axis), and cytotoxicity (secondary axis), assayed by LDH, from culture media of the treated spheroids. Results expressed as mean ± SD (*n* = 3). The letters a, b and c indicate a statistically significant difference at *p* < 0.01. Bars with no common letters are significantly different.

We evaluated the cytotoxic effects of the nurP28 and DTX monotherapies as well as their combination on MCF7 spheroids. Herein, we show the bright-field images of MCF7 spheroids exposed to different treatments (Figure 6A) with their corresponding monitored glucose concentrations and cytotoxicity values, assayed by LDH, in culture media (Figure 6B), as reference metabolic activity at various time points.

We observed morphological differences in the treated spheroids. In the control (i.e., spheroids not exposed to DTX or nurP28), we observed a well-defined growth-corona during the entire duration of the experiment. Similarly, a growth-corona was observed in the spheroids treated with 3 and 300 ng/mL nurP28. On the other hand, the spheroids exposed to DTX did not exhibit a growth-corona after 72 h of treatment.

These visual and qualitative observations were consistent with the values of glucose consumption and cytotoxicity observed in this experimental set. We did not observe significant differences in both glucose concentrations and cytotoxicity values in the first 72 h of culture among the treatments. However, after 120 h of culture, we did observe significant differences. Cytotoxicity was significantly higher in the group of spheroids treated with 3 ng/mL nurP28 + 1 µM DTX (16.8%) than the rest of the treatments. Albeit, the untreated control spheroids were significantly lower regarding the different treatments (5.2%).

**Figure 7:**
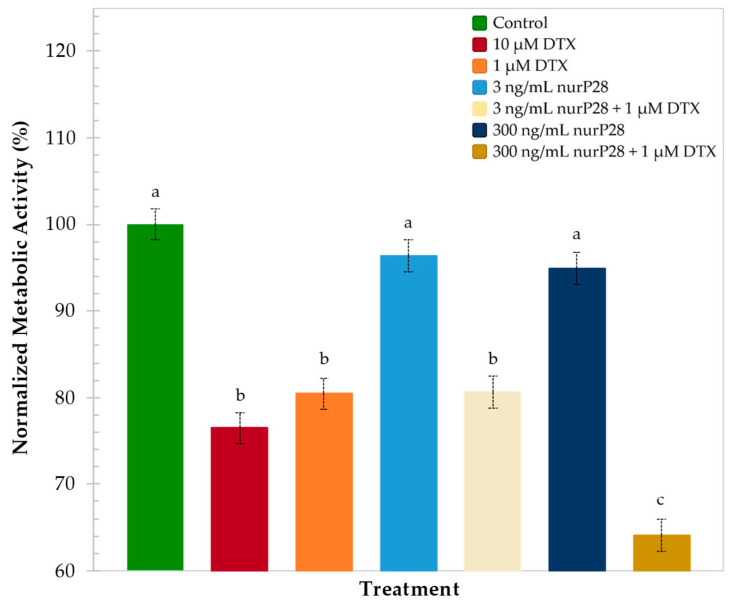
Normalized metabolic activity of breast cancer MCF7 spheroids after 120 h of exposure to the nurP28 peptide alone and in combination with DTX. Results expressed as mean ± SD (*n* = 3). The letters a, b, and c indicate a statistically significant difference at *p* < 0.01. Bars with no common letters are significantly different.

In contrast, glucose concentration was significantly higher in the spheroids treated with DTX (i.e., lower glucose consumption) than in the untreated control spheroids. Moreover, it remained statistically similar in the untreated control spheroids and the spheroids treated with nurP28. This suggests that treatment with nurP28 did not affect the glucose consumption rate in the spheroids during the first 120 h of exposure, whereas treatment with DTX alone and in combination with nurP28 did. Hence, we evaluated the metabolic activity of the spheroids after 120 h of treatment (Figure 7).

The comparative analysis of metabolic activities observed in each treatment group yielded three different response groups. The untreated spheroids (i.e., control) and spheroids exposed to 300 or 3 ng/mL nurP28 exhibited a statistically similar metabolic rate, which was higher than that observed in the spheroids exposed to DTX.

A similar trend was observed in the spheroids exposed to 1 µM DTX (80.4% nMA), 10 µM DTX (76.5% nMA), and 3 ng/mL nurP28 + 1 µM DTX (80.6% nMA).

Remarkably, the spheroids exposed to a lower concentration of DTX in combination with nurP28 (i.e., 300 ng/mL nurP28 + 1 µM DTX) exhibited the lowest metabolic activity (64.1% nMA). Interestingly, the effect of the nurP28 peptide was more potent in 3D spheroids exposed to 300 ng/mL nurP28 + 1 µM DTX (64.1% nMA) than in 2D monolayers exposed to the same treatment (83.0% nMA).

Note that this combination outperformed the highest dose of DTX tested (i.e., 10 µM).

## 3. Discussion

In this study, we rationally designed a new-to-nature peptide, nurP28, derived from the structure of a native peptide naturally found in zein. We designed our peptide based on factors such as polarity and charge because bioactivity can be enhanced through some sequence modifications, mainly those related to the amphipathic nature and net positive charge, which contribute to the disruption of cancer cell membranes and the entry of cytolytic peptides [38].

Moreover, we anticipated that altering the polarity by increasing the number of arginine residues in our zein-derived nurP28 peptide would increase its solubility and cell penetration efficiency. In particular, the insertion of arginine residues in peptides favors their initial electrostatic interaction with membranes and enhances their cell penetration efficiency [39,40,41]. Therefore, adding arginine residues conferred these properties to our nurP28 peptide.

Like other peptides, nurP28 is susceptible to degradation by proteolytic aminopeptidases or carboxypeptidases [42]. Studies have reported that the proteolytic stability of peptides can be increased by blocking the N- or C-terminus ends [43,44,45]. Thus, we added acetyl and amide groups at the N- and C-terminus for capping. These are two of the most common and reliable strategies for stabilizing peptides of almost any type [46].

Recent reports have demonstrated that positively charged peptides can disrupt the cell membrane of mammalian cells and induce cytotoxic effects [47,48]. However, cytotoxic effects are often nonspecific and may target noncancerous cells, such as macrophages or fibroblasts [49,50]. Therefore, it is important to identify peptides and concentration windows that may be useful for therapeutically treating tumors without affecting “healthy” cells [51].

Polyarginine peptides, such as nurP28, have been used in vitro and in vivo to enhance the cellular uptake of various drugs [41]. For instance, Karavasili et al. observed that the cytotoxic effect and cellular uptake of doxorubicin increased when administered in combination with Ac-(RADA)4-CONH2-DOX peptide than when administered alone [52].

Thus, our experimental strategy to explore the use of nurP28 consisted of first establishing its cytotoxicity in BJ cells (human fibroblasts), a human cell line commonly used in toxicological and pharmacological assays [53,54], and then exploring its role as an enhancer (chemosensitizer) of the therapeutic effect of DTX in two different culture systems of breast cancer (monolayer and spheroids).

In the cytocompatibility experiment, we included positive controls (i.e., 10 and 1 µM DTX) and negative controls (i.e., without DTX or nurP28). In this experimental setting, we did not observe any evidence of nurP28 cytotoxicity in the tested concentrations (3 and 300 ng/mL). However, we did observe the cytotoxicity of 10 µM DTX. Additionally, after 48 h of treatment, the combination therapies comprising 300 or 3 ng/mL + 1 µM DTX induced metabolic activity similar to that in the unexposed controls.

In our study, we used MCF7 monolayers and spheroids to compare the cytotoxicity of DTX alone, nurP28, and combination therapies of nurP28 and DTX. Monolayer cultures continue to be the widely used in vitro model for evaluating “anticancer” activity [55]. However, according to extensive literature evidence, spheroid cultures (and other 3D models) recreate tumor niches with higher fidelity than 2D cultures [56,57,58].

Cancer spheroids have become an interesting approach to evaluate candidate drugs [59] and a suitable in vitro platform to assay drug penetration and metabolism in 3D tumor microtissues [60]. Hence, the use of spheroids has been recently added as an approach to evaluate the “anticancer” activity of peptides [61,62].

Our results showed that nurP28 alone is not cytotoxic to breast cancer cell monolayers and spheroids at the evaluated concentrations. Surprisingly, nurP28 greatly enhances the cytotoxic effects of DTX in both breast cancer monolayer and spheroid cultures when administered in combination with 1 µM DTX.

The cytotoxic effect of combination therapy with 1 µM DTX + 3 ng/mL nurP28 was statistically similar to that of 10 µM DTX in the MCF7 monolayer culture. This suggested that nano doses of nurP28, which were noncytotoxic to human fibroblasts, could be used to reduce the doses of DTX required for breast cancer therapy.

In our experiments with MCF7 monolayers, nurP28 reduced the dose of DTX needed to decrease the metabolic activity and viability of cancer cells by one order of magnitude. In addition, the combination of nurP28 and 1 µM DTX could kill twice as many cancer cells as 1 µM DTX alone in MCF7 monolayers after 48 h of exposure.

The chemo-sensitizing effects of nurP28 were also apparent in the spheroid cultures. However, as expected, the time required to achieve the same cytotoxic effect in 2D and 3D cultures varied greatly. The diffusive transport of substances is more efficient in monolayer culture than in the spheroid core, where the diffusive transport is limited [57]. Consistently, none of the treatments caused a measurable decrease in the metabolic activity or cytotoxicity of spheroids during the first 72 h of exposure.

In this regard, the decreased rate of glucose consumption in spheroids treated with the combination of DTX and nurP28 signified the cytotoxic effect of the treatment only after 120 h of exposure. In particular, treatment with 10 µM DTX, 1 µM DTX, and the combination of 300 ng/mL nurP28 and 1 µM DTX, resulted in a statistically equivalent decrease in the metabolic activity of the spheroids. Notably, the time required to achieve the same cytotoxic effect in 2D culture was much less than that required in 3D culture; for instance, we observed signs of DTX cytotoxicity in MCF7 monolayers after 48 h of exposure.

We also assessed the LDH activity, as an indicator of cell death [63] and cell membrane damage [64], in 3D breast cancer spheroids. The degree of cell membrane damage was significantly higher in the spheroids treated with nurP28 alone than in untreated controls, while similar to that in the spheroids treated with DTX only. This suggests that nurP28 is capable of causing cell membrane damage on its own, even at the low dosage tested. A preliminary set of immunostaining experiments has also revealed a higher expression of markers of apoptosis (caspase 3 and caspase 8) in 2D monolayers of MCF7 cells exposed to nurP28 than in untreated controls (Supplementary Figure S3).

Remarkably, the superior cytotoxicity associated with the combination therapy of 300 ng/mL nurP28 and 1 µM DTX was more evident in spheroids than in monolayers. Compared with 10 µM DTX, this combination therapy was significantly more effective in spheroids. The combination therapy decreased the metabolic rate of spheroid cultures by nearly 40% compared with the control. Moreover, its cytotoxicity was nearly three times higher than in the untreated control spheroids. In comparison, 1 and 10 µM DTX decreased the metabolic rate by only 20% after 120 h of exposure. The cytotoxicity of the combination therapy of 3 ng/mL nurP28 and 1 µM DTX was equivalent but not higher than that of 10 µM DTX in monolayer culture.

Our results suggested that nurP28 can penetrate and facilitate the cytotoxic action of DTX in both 2D and 3D tissues. Chemo-sensitizing peptides capable of selectively enhancing the cytotoxic effects of “anticancer” agents have been reported [38,62]. Nevertheless, to our proven knowledge, this is the first report describing a zein-derived peptide capable of enhancing the effects of potent anticancer drugs [35]. However, further studies for establishing the precise mechanism by which nurP28 acts as a chemo-sensitizer remain outside the scope of this work.

## 4. Materials and Methods

### 4.1. De Novo Peptide Design

We conducted bioinformatics analysis to identify possible bioactive zein-derived peptides. Based on this analysis, we selected the protein sequence of 22-kDa alpha-zein reported in UniProt (accession Q00919_MAIZE).

We analyzed the FASTA format sequence of this protein using the TMHMM Server v.2.0 [36] (Figure S1) and PROTEUS Structure Prediction Server 2.0 [37] (Figure S2) to predict the transmembrane helices. After selecting the potential transmembrane sequence, we modified the amino acids Ser8, Ser10, Val11, and Ser12 to arginine residues in the native sequence.

Subsequently, we obtained a PDB structural model for the resulting transmembrane peptide using the I-TASSER server [65]. The resulting model was further processed using PyMOL version 2.2.2 [66].

### 4.2. Peptide Synthesis

The nurP28 (purity ≥ 98%) was synthesized by Applied Biological Materials Inc. (Richmond, Canada). The lyophilized peptide was stored at −20 °C until use. The peptide (1 mg/mL) was dissolved in phosphate-buffered saline (PBS; 75 mM, pH 7.4) and used as a stock for the experiments reported herein.

### 4.3. Cell Lines and Culture

MCF7 (ATCC HTB-22, Rockville, FL, USA) and BJ (ATCC CRL-2522, Rockville, FL, USA) cell lines were used. MCF7 cells were cultured in Dulbecco’s Modified Eagle Medium and BJ cells were cultured in Eagle’s minimum essential medium. Culture media were supplemented with 10% fetal bovine serum and 1% antibiotic–antimycotic solution (Gibco 1524006; Waltham, MA, USA). The cells were cultured in 25-cm^2^ cell culture flasks (Corning 430639; Corning, NY, USA) in a humidified atmosphere of 5% CO_2_ at 37 °C. The medium was replaced every 2 days during cell maintenance. The cells were detached using trypsin–EDTA solution (Gibco R001100; Waltham, MA, USA) when 70% confluency was reached and either passaged or used for experiments. Only cells with a passage number lower than 20 were used for experiments.

### 4.4. Treatments

We used PBS (75 mM, pH 7.4; Merck 806552, Darmstadt, Germany) as a negative control for experiments. For positive response control, 1 and 10 µM DTX (Merck 01885, Darmstadt, Germany) were used. These DTX dosing regimens (1 and 10 µM) have been tested previously in cytotoxicity assays using cancer cell monolayers [67,68]. We also used 3 and 300 ng/mL nurP28 peptide alone and in combination with 1 µM DTX.

### 4.5. Fabrication of 3D Spheroids

When 80% confluency was reached, 5 × 10^5^ MCF7 cells per well were transferred to ultra-low attachment microplates (Corning 4520; Corning, NY, USA) to induce spheroid formation [69]. A working volume of 200 µL of culture medium was added and maintained in each well. After 3-day incubation in static conditions at 37 °C in a humidified atmosphere containing 5% CO_2_, spheroids were checked for structure solidness using a microscope and further treated.

### 4.6. Glucose Consumption Monitoring of Spheroids

Medium samples (4 µL) were collected from each treatment well containing spheroids to determine the glucose concentration at 0, 24, 72, and 120 h using an Accu-Check Active device (Roche Diagnostics, Monza, Italy), according to the manufacturer’s instructions.

### 4.7. Lactate Dehydrogenase Cytotoxicity Assay

We performed the fluorometric LDH-cytotoxicity assay (Abcam, Boston, MA, USA ab197004) to assess the degree of cell death on spheroids in response to the exposure to our treatments. We collected medium samples (5 µL) from wells containing spheroids corresponding to the treatments. According to the manufacturer’s instructions, we assayed our samples plus negative and positive controls to determine the cytotoxicity percent. We used a Synergy HT microplate reader (Agilent, Santa Clara, CA, USA) to obtain the 535 and 587 nm fluorescence readings corresponding to the wavelengths for excitation and emission, respectively.

### 4.8. Microscopy and Imaging

The changes in the size and shape of the MCF7 spheroids were monitored using an inverted optical Axio Observer.Z1 microscope (Zeiss, Göttingen, Germany) in a bright field. Morphological changes after treatments were observed at 0, 24, and 48 h. In addition, the qualitative differences in the viability of cells or spheroids subjected to different treatments were investigated using a live/dead assay kit (Invitrogen, Carlsbad, CA, USA). Samples were prepared according to the manufacturer’s instructions and imaged under fluorescence illumination using a Colibri.2 LED illumination and an Apotome.2 system (Zeiss) coupled with an Axio Observer.Z1 microscope (Zeiss). LED illuminator intensity and exposure time were specifically set at 30% and 50 ms for FITC and 60% and 800 ms for EtHD1 channels. Micrographs were obtained using a 10× objective.

### 4.9. Metabolic Activity Assays

In 2D cytocompatibility and chemo-sensitizing assays, 2 × 10^4^ BJ and 1 × 10^4^ MCF7 cells per well were cultured for 48 h in 48- and 96-well plates, with final volumes of 500 and 250 µL, respectively. The cells were treated after 24 h in the experimental plates. At 48 h, PrestoBlue reagent protocol (ThermoFisher A13262, Waltham, MA, USA) was used to determine the rate of metabolic activity. Treated cells were incubated in 1× PrestoBlue reagent for 2 h. Fluorescence was read at 525/615 nm excitation/emission bandwidth using a Synergy HT microplate reader (Agilent, Santa Clara, CA,, USA). The average reading obtained for cell-free wells was subtracted from the readings obtained for samples for the calculation of final fluorescence values. Finally, the metabolic activity was calculated using the following formula:

% Metabolic activity = corrected fluorescence of treated cells/corrected fluorescence of controls × 100(1)

### 4.10. Statistical Analysis

Independent experiments (*n* = 3) were conducted in triplicate. Raw data of experiments (Tables S1–S4) were processed as mean ± SD. Significant differences among the treatment solutions were determined using ANOVA with Dunnett’s multiple comparison post hoc test (α = 0.01), with the vehicle as the control. These analyses were performed using XLSTAT 2021.1.1 (Addinsoft, New York, NY, USA).

## 5. Conclusions

We introduced nurP28, a new-to-nature peptide derived from a naturally occurring sequence found in α-zein, a major constitutive protein of maize seeds. We evaluated the cytotoxicity of nurP28, alone and in combination with DTX, in fibroblast monolayers, breast cancer monolayers, and spheroids. Our results indicated that nurP28 is not cytotoxic to human fibroblasts or cancer cells.

Nevertheless, when combined with 1 µM DTX, 3 ng/mL nurP28 induced equivalent (in MCF7 monolayers) and higher (in MCF7 spheroids) cytotoxic effects than 10-fold higher doses of DTX alone (10 µM DTX). These findings show that nurP28 can significantly enhance the penetration of DTX into breast cancer spheroids, and they suggest that this zein-derived peptide should be further explored as a chemosensitizer for breast cancer treatment with DTX.

The addition of a nanomolar dose of nurP28 may effectively decrease the DTX dose required to achieve a relevant therapeutic effect, while possibly diminishing the occurrence of the side effects of targeting cancer therapies. Further studies are needed to establish the exact mechanisms behind these findings, to assess their clinical relevance in animal models, and to establish if the same effects would be observed with nurP28 combinations with other targeting cancer drugs or in other tumor types.

## Data Availability

The data presented in this study are available in the main text of the manuscript and in supplementary material.

## Supplementary Materials

The following supporting information can be downloaded at: https://www.mdpi.com/article/10.3390/molecules27092824/s1, Video S1. Secondary structure of nurP28 peptide; Figure S1. Transmembrane trend probability for 22-KDa α-zein amino acid sequence obtained by TMHMM Server v.2.0; Figure S2. Predicted transmembrane and secondary structures for α-zein, obtained by PROTEUS Structure Prediction Server 2.0; Figure S3. Breast cancer MCF7 monolayers after 48 h of exposure to nurP28. (A) Bright field and immunostaining images for caspases 3 and 8 in untreated (control) and treated cells. Cell nuclei appear in blue. Positive cells for caspases 3 and 8 appear green/yellow; (B) Assessment of cytoxicity from culture media of untreated and treated monolayers as determined in LDH activity assays. Results expressed as mean ± SD (*n* = 3). The letters a and b indicate a statistically significant difference at *p* < 0.01. Bars with no common letters are significantly different. Magnification: 10x. Scale bar: 200 µM; Table S1. Fluorescence readings (FU) and normalized metabolic activities (NMA %) of BJ fibroblast cells; Table S2. Fluorescence readings (FU) and normalized metabolic activities (NMA %) of MCF7 monolayers; Table S3. Fluorescence readings (FU) and normalized metabolic activities (NMA %) of spheroids at 120 h; and Table S4. Glucose readings of spheroids culture media at 0, 24, 72 and 120 h.

## Sample Availability

Samples of the compound are available from the authors.
