# Supplementary material for: nurP28, a New-to-Nature Zein-Derived Peptide, Enhances the Therapeutic Effect of Docetaxel in Breast Cancer Monolayers and Spheroids
Source: Molecules. 2022 Apr 29;27(9):2824. doi: 10.3390/molecules27092824 (PMC9105272; doi:10.3390/molecules27092824)
Supplement: Supplementary file 1 [file molecules-27-02824-s001.zip › molecules-1663330 SI/molecules-1663330 SI.pdf]

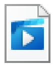

Secondary Structure
of nurP28 peptide.mp

Video S1. Secondary structure of nurP28 peptide.

The 22-KDa α-zein sequence analysis by TMHMM Server v.2.0 resulted in only a peak for transmembrane trend probability (Figure S1). This peak, located between the amino acids 6-25, showed a probability value > 0.9. A short region composed by the first five amino acid of the α-zein sequence was predicted to be located inside the cell. The remaining 26 to 266 amino acids were located outside the cell with a probability ~ 1.0.

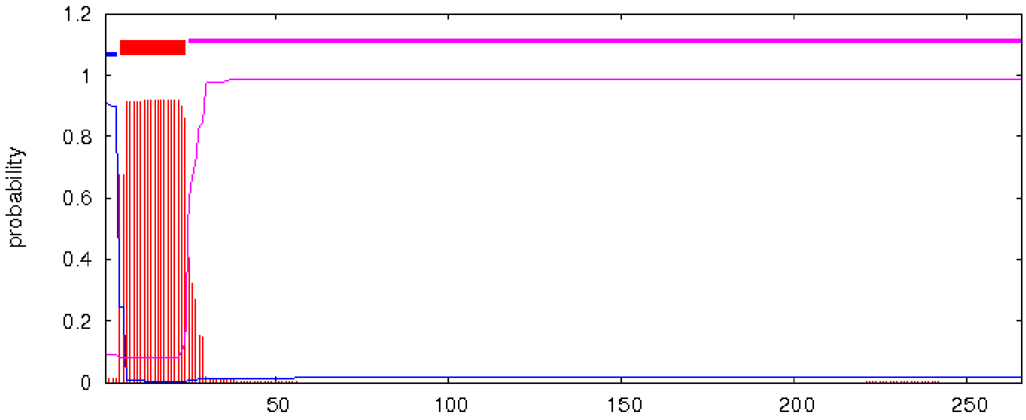

Figure S1. Transmembrane trend probability for 22-KDa α-zein amino acid sequence obtained by TMHMM Server v.2.0

An additional amino acid analysis was performed on PROTEUS Structure Prediction Server 2.0 (Figure S2).

```
  1   MATKILALLALLSLSVSATTAFIIPQCSLAPNAIIPQFLPSVTSMGIEHPIVQAYRLQQA 60
      CCHHHTTTTTTTTTTTTTTTTTTTTCCCCCCCCCCCCHHHCCCCCCCCCCCCHHHHHHHH
      957899999999999999999999977766765556654455555555886545999999

 61   LAASVLQQPFAQLQQQSLAHLTIQTIATQLEQQFVPALSQLAAVNPVSYLQQQMLASNPL 120
      HHHHHHHHHHHHHHHHHHHHHHHHHHHHHHHHHHHHHHHHHHHHHHHHHHHHHHHHHHHH
      999999757999976799999999999999999999999999899999999996445699

121   ALANTAAYQQQLQLQQFLPALSQLARVNPATYLQQQLLSSSPLAVGNAATYLQQQLLQQ 180
      HHHHHHHHHHHHHHHHHHHHHHHHHHHHHHHHHHHHHHCCCCCCCCCHHHHHHHHHHHHH
      999999999999999999999999999999999999967888865666689999999999

181   IVPALSQLVVANPTAYLQQLLPFNQLDVANSAAYLQQRQQLLNPLAAANPLVAAFLQQQQ 240
      HHHHHHHHHHHHHHHHHHHHHHCCCHHHHHHHHHHHHHHHHHHCCHHHHHHHHHHHHHHH
      999999999999788999999956648999999999999999845899999999999999

241   FLPYNQISLMNLALSRQQPIVGGAIF 266
      HHHHHHHHHHHHHHHCCCCCCCCCCC
      85799999976656556766778888
```

```
H = Helix
E = Beta Strand
C = Coil
T = Membrane helix
B = Membrane strand
S = Signal peptide
c = Cleavage site
Line 1 = sequence (single letter IUPAC code, 60 characters per line)
Line 2 = secondary structure (H, E or C)
Line 3 = confidence score (0-9, 0 = low, 9 = high)
```

Figure S2. Predicted transmembrane, and secondary structures for α-zein, obtained by PROTEUS Structure Prediction Server 2.0

The results exhibited a sole transmembrane region conformed by 20 amino acid within the α-zein sequence, located between the amino acids 6 to 25. Leucine (30%), alanine (20%), valine (5%), phenylalanine (5%), isoleucine (10%), and proline (5%), all hydrophobic aminoacidic, composed 75% of the sequence. Serine (15%), and threonine (10%), polar amino acids, conformed the remaining part of the region. No charged amino acid was found within such region. Additionally, helix-forming trends were observed between the amino acids 53 to 158, 168 to 202, 206 to 223, and 226 to 255.

Table S1. Fluorescence readings (FU) and normalized metabolic activities (NMA %) of BJ fibroblast cells.

Treatment	FU	NMA %
Control	2986	101.051
Control	2976	100.701
Control	2960	100.175
Control	2955	100.000
Control	2950	99.825
Control	2948	99.772
Control	2943	99.586
Control	2934	99.299
Control	2929	99.124
DTX 10 μM	2339	79.159
DTX 10 μM	2350	79.510
DTX 10 μM	2370	80.210
DTX 10 μM	2401	81.261
DTX 10 μM	2417	81.786
DTX 10 μM	2417	81.786
DTX 10 μM	2422	81.961
DTX 10 μM	2474	83.713
DTX 10 μM	2531	85.639
DTX 1 μM	2557	86.515
DTX 1 μM	2603	88.091
DTX 1 μM	2639	89.317
DTX 1 μM	2774	93.870
DTX 1 μM	2803	94.860
DTX 1 μM	2827	95.671
DTX 1 μM	2842	96.177
DTX 1 μM	2867	97.023
DTX 1 μM	2933	99.266
nurP28 3 ng/mL	2771	93.763
nurP28 3 ng/mL	2810	95.096
nurP28 3 ng/mL	2831	95.797
nurP28 3 ng/mL	2851	96.497
nurP28 3 ng/mL	2867	97.038
nurP28 3 ng/mL	2914	98.599
nurP28 3 ng/mL	2939	99.475
nurP28 3 ng/mL	3002	101.576
nurP28 3 ng/mL	3053	103.327
nurP28 3 ng/mL + DTX 1 μM	2635	89.155
nurP28 3 ng/mL + DTX 1 μM	2732	92.469
nurP28 3 ng/mL + DTX 1 μM	2784	94.221
nurP28 3 ng/mL + DTX 1 μM	2862	96.848
nurP28 3 ng/mL + DTX 1 μM	2862	96.848
nurP28 3 ng/mL + DTX 1 μM	2872	97.198
nurP28 3 ng/mL + DTX 1 μM	2883	97.548
nurP28 3 ng/mL + DTX 1 μM	2888	97.723
nurP28 3 ng/mL + DTX 1 μM	2893	97.898
nurP28 300 ng/mL	3121	105.604
nurP28 300 ng/mL	3172	107.356
nurP28 300 ng/mL	3183	107.706
nurP28 300 ng/mL	3224	109.107
nurP28 300 ng/mL	3229	109.282
nurP28 300 ng/mL	3255	110.158
nurP28 300 ng/mL	3400	115.061
nurP28 300 ng/mL	3457	116.988
nurP28 300 ng/mL	3617	122.417
nurP28 300 ng/mL + DTX 1 μM	2547	86.201
nurP28 300 ng/mL + DTX 1 μM	2625	88.818
nurP28 300 ng/mL + DTX 1 μM	2679	90.657
nurP28 300 ng/mL + DTX 1 μM	2743	92.820
nurP28 300 ng/mL + DTX 1 μM	2815	95.271
nurP28 300 ng/mL + DTX 1 μM	2872	97.198
nurP28 300 ng/mL + DTX 1 μM	2950	99.825
nurP28 300 ng/mL + DTX 1 μM	2965	100.350
nurP28 300 ng/mL + DTX 1 μM	2976	100.701

Table S2. Fluorescence readings (FU) and normalized metabolic activities (NMA %) of MCF7 monolayers.

Treatment	FU	NMA %
Control	3818	107.882
Control	3548	100.253
Control	3478	98.275
Control	3574	100.988
Control	3805	107.515
Control	3577	101.073
Control	3383	95.591
Control	3455	97.625
Control	3213	90.787
10μM DTX	2850	80.530
10μM DTX	2859	80.770
10μM DTX	3349	94.616
10μM DTX	3092	87.368
10μM DTX	3316	93.698
10μM DTX	2871	81.124
10μM DTX	3071	86.775
10μM DTX	2862	80.869
10μM DTX	2850	80.530
1μM DTX	3039	85.871
1μM DTX	2755	77.846
1μM DTX	3436	97.088
1μM DTX	3295	93.104
1μM DTX	3239	91.522
1μM DTX	3275	92.539
1μM DTX	3236	91.437
1μM DTX	3658	103.361
1μM DTX	3216	90.872
3ng/mL nurP28	3675	103.842
3ng/mL nurP28	3664	103.531
3ng/mL nurP28	3737	105.594
3ng/mL nurP28	4023	113.675
3ng/mL nurP28	3737	105.594
3ng/mL nurP28	3751	105.989
3ng/mL nurP28	3739	105.650
3ng/mL nurP28	3892	109.973
3ng/mL nurP28	3847	108.702
3ng/mL nurP28 + 1μM DTX	3259	92.087
3ng/mL nurP28 + 1μM DTX	3098	87.538
3ng/mL nurP28 + 1μM DTX	3277	92.596
3ng/mL nurP28 + 1μM DTX	3255	91.974
3ng/mL nurP28 + 1μM DTX	3217	90.900
3ng/mL nurP28 + 1μM DTX	3300	93.246
3ng/mL nurP28 + 1μM DTX	3130	88.442
3ng/mL nurP28 + 1μM DTX	3441	97.230
3ng/mL nurP28 + 1μM DTX	3188	90.081
300ng/mL nurP28	3746	105.848
300ng/mL nurP28	3960	111.895
300ng/mL nurP28	3858	108.998
300ng/mL nurP28	4060	114.720
300ng/mL nurP28	3780	106.809
300ng/mL nurP28	3763	106.328
300ng/mL nurP28	3515	99.321
300ng/mL nurP28	3708	104.774
300ng/mL nurP28	3621	102.316
300ng/mL nurP28 + 1μM DTX	2758	77.931
300ng/mL nurP28 + 1μM DTX	3008	84.995
300ng/mL nurP28 + 1μM DTX	2755	77.846
300ng/mL nurP28 + 1μM DTX	2809	79.372
300ng/mL nurP28 + 1μM DTX	2850	80.530
300ng/mL nurP28 + 1μM DTX	2859	80.785
300ng/mL nurP28 + 1μM DTX	2971	83.949
300ng/mL nurP28 + 1μM DTX	3107	87.792
300ng/mL nurP28 + 1μM DTX	3332	94.150

Table S3. Fluorescence readings (FU) and normalized metabolic activities (NMA %) of spheroids at 120 h.

Treatment	FU	NMA %
Control	3550	99.485
Control	3832	107.389
Control	3574	100.167
Control	3384	94.852
Control	3384	94.852
Control	3394	95.124
Control	3467	97.168
Control	3739	104.800
Control	3788	106.163
10μM DTX	2597	72.774
10μM DTX	2665	74.682
10μM DTX	2704	75.772
10μM DTX	2665	74.682
10μM DTX	2650	74.273
10μM DTX	2572	72.093
10μM DTX	2572	72.093
10μM DTX	3034	85.039
10μM DTX	3097	86.811
1μM DTX	2665	74.682
1μM DTX	2650	74.273
1μM DTX	3229	90.491
1μM DTX	2815	78.907
1μM DTX	2849	79.861
1μM DTX	3214	90.082
1μM DTX	2898	81.224
1μM DTX	2956	82.859
1μM DTX	2553	71.548
3ng/mL nurP28	3457	96.896
3ng/mL nurP28	3540	99.213
3ng/mL nurP28	3326	93.216
3ng/mL nurP28	3360	94.170
3ng/mL nurP28	3316	92.944
3ng/mL nurP28	3355	94.034
3ng/mL nurP28	3438	96.351
3ng/mL nurP28	3535	99.076
3ng/mL nurP28	3613	101.257
3ng/mL nurP28 + 1μM DTX	2786	78.089
3ng/mL nurP28 + 1μM DTX	2811	78.770
3ng/mL nurP28 + 1μM DTX	2990	83.813
3ng/mL nurP28 + 1μM DTX	3029	84.903
3ng/mL nurP28 + 1μM DTX	2723	76.317
3ng/mL nurP28 + 1μM DTX	2893	81.087
3ng/mL nurP28 + 1μM DTX	2961	82.995
3ng/mL nurP28 + 1μM DTX	2864	80.270
3ng/mL nurP28 + 1μM DTX	2830	79.316
300ng/mL nurP28	3273	91.717
300ng/mL nurP28	3258	91.308
300ng/mL nurP28	3073	86.130
300ng/mL nurP28	3122	87.492
300ng/mL nurP28	3093	86.675
300ng/mL nurP28	3676	103.028
300ng/mL nurP28	3773	105.754
300ng/mL nurP28	3584	100.439
300ng/mL nurP28	3627	101.666
300ng/mL nurP28 + 1μM DTX	2067	57.919
300ng/mL nurP28 + 1μM DTX	2105	59.010
300ng/mL nurP28 + 1μM DTX	2426	68.004
300ng/mL nurP28 + 1μM DTX	2164	60.645
300ng/mL nurP28 + 1μM DTX	1974	55.330
300ng/mL nurP28 + 1μM DTX	2402	67.323
300ng/mL nurP28 + 1μM DTX	2373	66.505
300ng/mL nurP28 + 1μM DTX	2514	70.457
300ng/mL nurP28 + 1μM DTX	2563	71.820

Table S4. Glucose readings of spheroids culture media at 0, 24, 72 and 120 hours.

	Glucose [mg·mL^{-1}]			
**Treatment**	**0 h**	**24 h**	**72 h**	**120 h**
Control	85	86	79	67
Control	86	86	78	62
Control	95	82	78	67
Control	84	86	81	75
Control	86	87	78	72
Control	83	79	81	70
Control	83	82	75	64
Control	89	87	80	73
Control	87	83	79	71
10μM DTX	85	80	81	83
10μM DTX	86	86	77	75
10μM DTX	79	81	78	78
10μM DTX	86	80	82	82
10μM DTX	86	84	83	84
10μM DTX	86	83	83	80
10μM DTX	86	85	78	75
10μM DTX	84	86	76	75
10μM DTX	80	84	78	78
1μM DTX	85	85	77	79
1μM DTX	86	83	79	76
1μM DTX	86	80	80	75
1μM DTX	80	77	80	73
1μM DTX	83	84	79	75
1μM DTX	85	81	78	76
1μM DTX	85	81	76	77
1μM DTX	86	81	84	78
1μM DTX	85	81	81	82
3ng/mL nurP28	85	85	77	66
3ng/mL nurP28	86	84	83	69
3ng/mL nurP28	84	82	76	69
3ng/mL nurP28	86	82	75	67
3ng/mL nurP28	86	81	79	69
3ng/mL nurP28	88	84	77	69
3ng/mL nurP28	85	85	76	71
3ng/mL nurP28	84	81	78	69
3ng/mL nurP28	83	82	76	68
3ng/mL nurP28 + 1μM DTX	85	85	86	87
3ng/mL nurP28 + 1μM DTX	86	88	85	90
3ng/mL nurP28 + 1μM DTX	86	88	87	81
3ng/mL nurP28 + 1μM DTX	86	88	80	78
3ng/mL nurP28 + 1μM DTX	85	84	82	77
3ng/mL nurP28 + 1μM DTX	87	78	79	78
3ng/mL nurP28 + 1μM DTX	84	80	78	77
3ng/mL nurP28 + 1μM DTX	85	78	81	79
3ng/mL nurP28 + 1μM DTX	82	82	82	80
300ng/mL nurP28	85	83	72	68
300ng/mL nurP28	86	81	78	64
300ng/mL nurP28	82	84	72	63
300ng/mL nurP28	86	81	73	67
300ng/mL nurP28	79	81	80	72
300ng/mL nurP28	82	84	77	71
300ng/mL nurP28	83	80	81	71
300ng/mL nurP28	86	84	77	73
300ng/mL nurP28	86	83	78	70
300ng/mL nurP28 + 1μM DTX	85	82	81	82
300ng/mL nurP28 + 1μM DTX	86	80	80	79
300ng/mL nurP28 + 1μM DTX	81	81	78	79
300ng/mL nurP28 + 1μM DTX	76	79	84	84
300ng/mL nurP28 + 1μM DTX	81	79	82	82
300ng/mL nurP28 + 1μM DTX	77	80	78	74
300ng/mL nurP28 + 1μM DTX	80	78	76	75
300ng/mL nurP28 + 1μM DTX	79	80	76	77
300ng/mL nurP28 + 1μM DTX	83	79	81	77
