# Supplementary figures and images for: nurP28, a New-to-Nature Zein-Derived Peptide, Enhances the Therapeutic Effect of Docetaxel in Breast Cancer Monolayers and Spheroids
Source: Molecules. 2022 Apr 29;27(9):2824. doi: 10.3390/molecules27092824 (PMC9105272; doi:10.3390/molecules27092824)

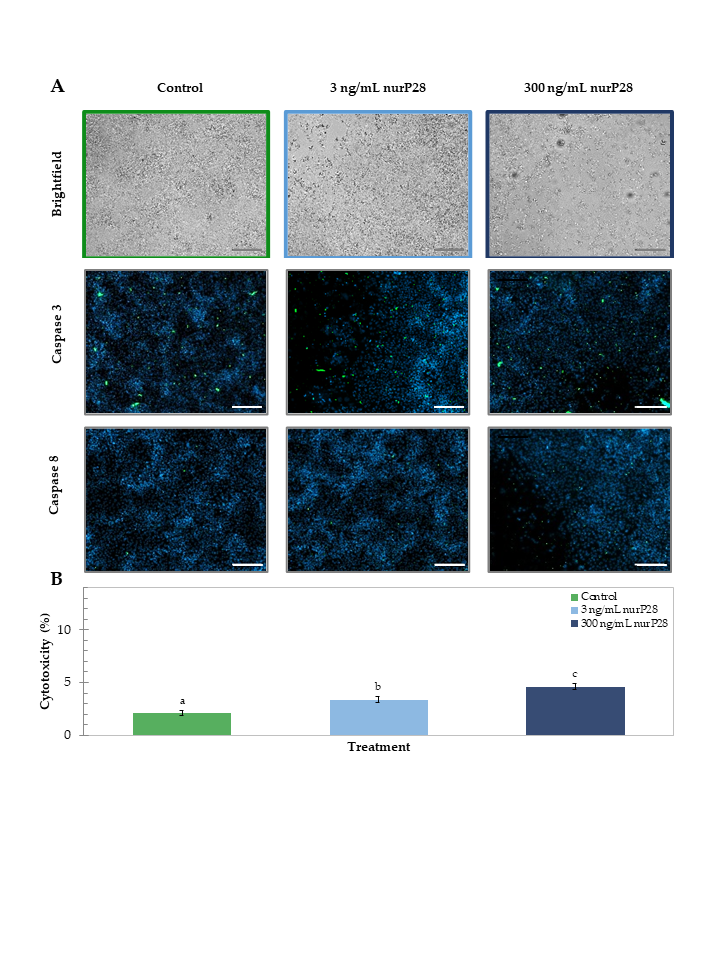

Supplement: Supplementary file 1 [file molecules-27-02824-s001.zip › molecules-1663330 SI/Supplementary Figure S3_rev.tif]
